# Management of Traumatized Teeth With Severely Calcified Canals and Minimally Invasive Access Cavity Using the AReneto® System: A Case Report

**DOI:** 10.7759/cureus.70298

**Published:** 2024-09-27

**Authors:** Pucha Sai Manaswini, Varun Prabhuji, Champa C, Srirekha A, Veena S Pai

**Affiliations:** 1 Department of Conservative Dentistry and Endodontics, The Oxford Dental College, Bangalore, IND

**Keywords:** areneto® system, augmented reality, calcified canals, digital dentistry, minimally invasive endodontics

## Abstract

Traumatic injuries to anterior teeth often lead to complications such as calcification and periapical lesions, which frequently necessitate root canal treatment. Traditional methods of preparing access cavities in these cases can compromise the tooth’s structural integrity, impacting long-term outcomes. With the advancements in minimally invasive techniques, guided endodontics has emerged as a critical innovation in modern dentistry. This article introduces the AReneto® system, developed by Roots to Cusps® Private Limited, which integrates augmented reality (AR) into endodontic procedures, enabling clinicians to enhance precision and preserve tooth structure.

A 24-year-old male presented to the Department of Conservative Dentistry and Endodontics with discolored upper front teeth, reporting a history of trauma four years earlier. Clinical and radiographic evaluation revealed canal calcification in tooth 21 and a periapical lesion in tooth 11. Both teeth were tender upon percussion, and the cold test elicited no response. Consequently, a diagnosis of pulpal necrosis with a periapical abscess was made for tooth 11, while tooth 21 was diagnosed with pulp canal obliteration and symptomatic apical periodontitis, necessitating root canal treatment for both teeth. Minimally invasive root canal treatment was performed using the AReneto® system for access opening. The canals were then cleaned, shaped, and subsequently obturated.

The AReneto® system represents a significant advancement in guided endodontics, integrating the latest technologies to improve patient outcomes and enhance the long-term prognosis of treated teeth by assisting in performing minimally invasive access openings.

## Introduction

Traumatic dental injuries frequently lead to complications such as calcifications and periapical lesions, necessitating root canal treatment [1]. Traditional access cavity preparation often compromises tooth strength, hindering treatment success [2].

A meticulously designed and executed access cavity is essential for treatment success, as it facilitates optimal canal negotiation, debridement, and obturation while minimizing the risk of procedural errors and subsequent endodontic complications [3].

To address this challenge, guided endodontics has emerged [4], utilizing 3D-printed templates generated from cone beam computed tomography (CBCT) and intraoral scans. This is particularly useful in cases of calcified canals [5]. Three-dimensional (3D)-guided templates are often more time-consuming and expensive, and they can complicate the operating area, making the procedure more challenging for the clinician [6].

Augmented reality (AR) is rapidly gaining popularity worldwide as it overlays information directly in front of the user’s eyes. By combining guided endodontics with AR, Roots to Cusps® Private Limited, based in Bangalore, India, developed the AReneto® system [7]. The AReneto® system displays preoperative virtual planning of the access cavity directly in front of the clinician’s eyes through AR glasses. This approach eliminates the need for constant shifting between the patient and the laptop screen. This use of AReneto® system with preoperative guide planning enhances efficiency in minimally invasive access cavity procedures [7].

This case report details the use of the AReneto® system for access cavity preparation in traumatized teeth. The AReneto® system aids the clinician in achieving optimal access to root canal treatment [7]. Ultimately, the goal is to improve long-term outcomes for patients with dental injuries.

## Case presentation

**Figure 1:**
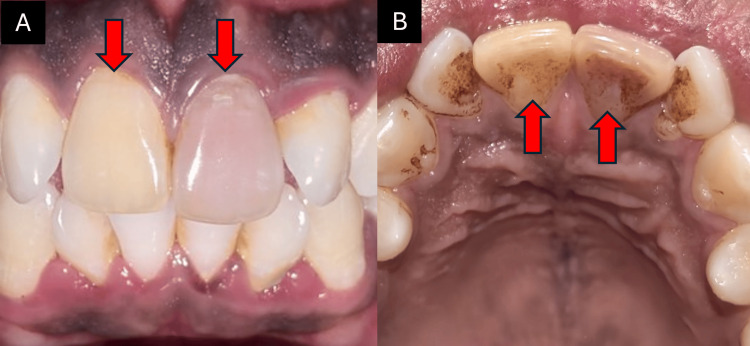
(A) Preoperative facial view and (B) preoperative incisal view

**Figure 2:**
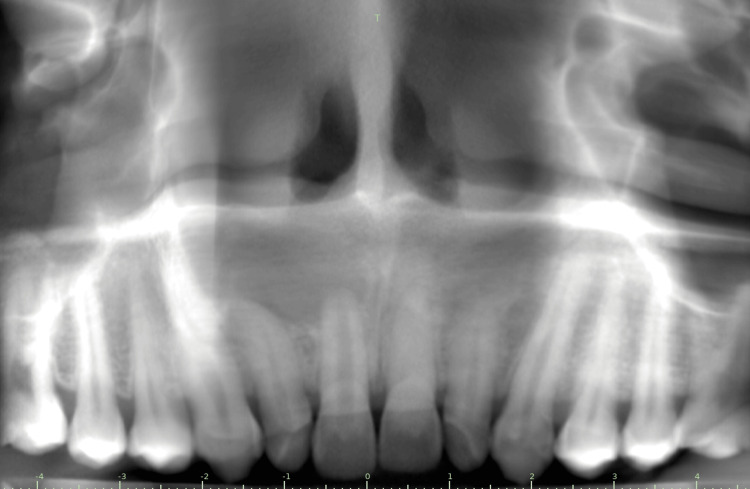
Preoperative radiograph

A 24-year-old male patient presented to the department with discolored upper front teeth (Figure 1) and a history of trauma four years ago. Clinical and radiographic evaluation revealed canal calcification in tooth 21 and a periapical lesion in tooth 11 (Figure 2). The cold test showed no response, indicating the need for root canal treatment. The patient was given two options: conventional root canal treatment followed by full coverage crowns or AR-guided minimally invasive endodontic approach through the incisal third followed by ceramic veneers using the AReneto® system. The patient opted for an AR-guided approach followed by ceramic veneers.

**Figure 3:**
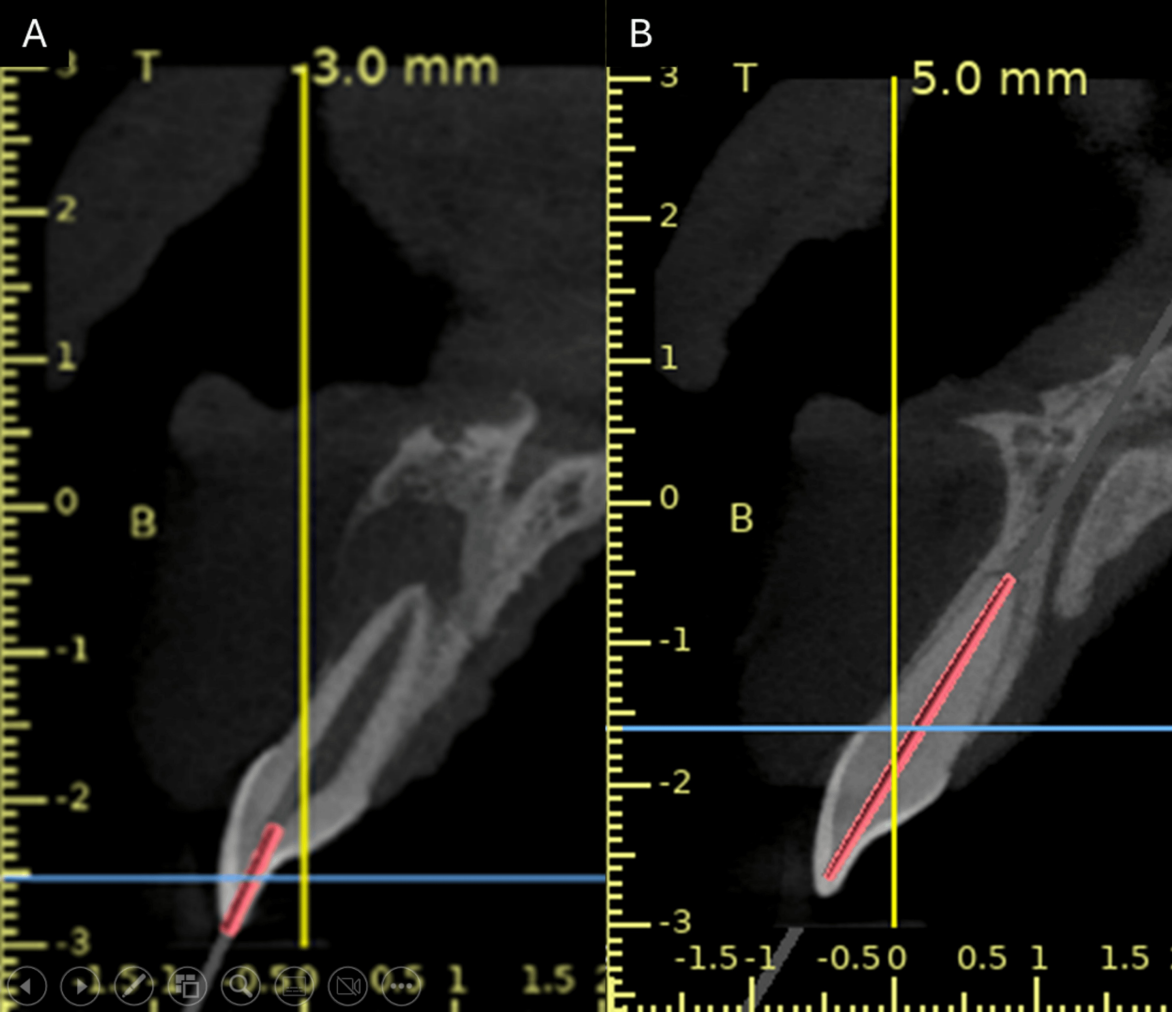
(A) Guide designing for tooth 11 and (B) guide designing for tooth 21

The patient underwent a cone beam computed tomography (CBCT) scan (CS9600, Carestream Health, Rochester, New York) to obtain the digital imaging and communications in medicine (DICOM) file. This DICOM file was transferred to 3D implant planning software (Blue Sky Bio, LLC, Libertyville, Illinois), where a guide for access cavity preparation was planned (Figure 3).

**Figure 4:**
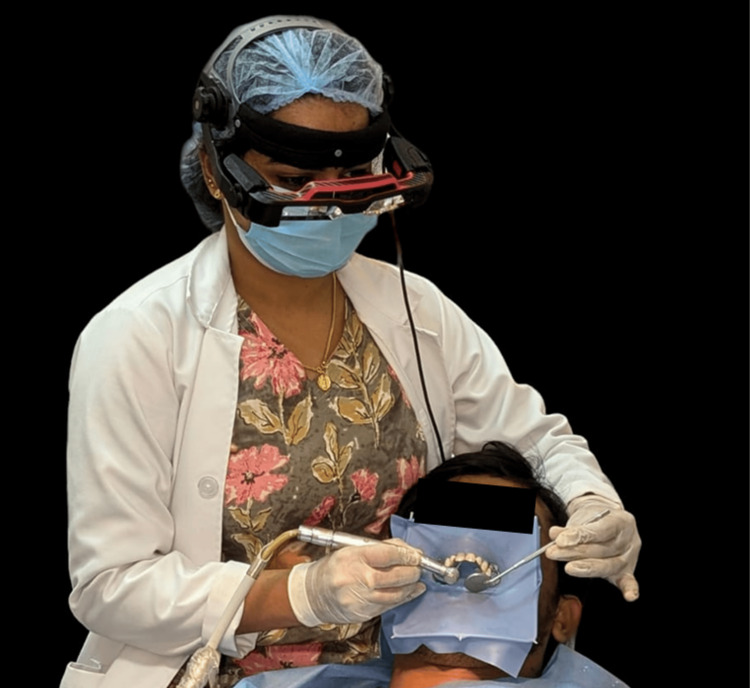
Clinician wearing the AR glasses AR: Augmented reality.

**Figure 5:**
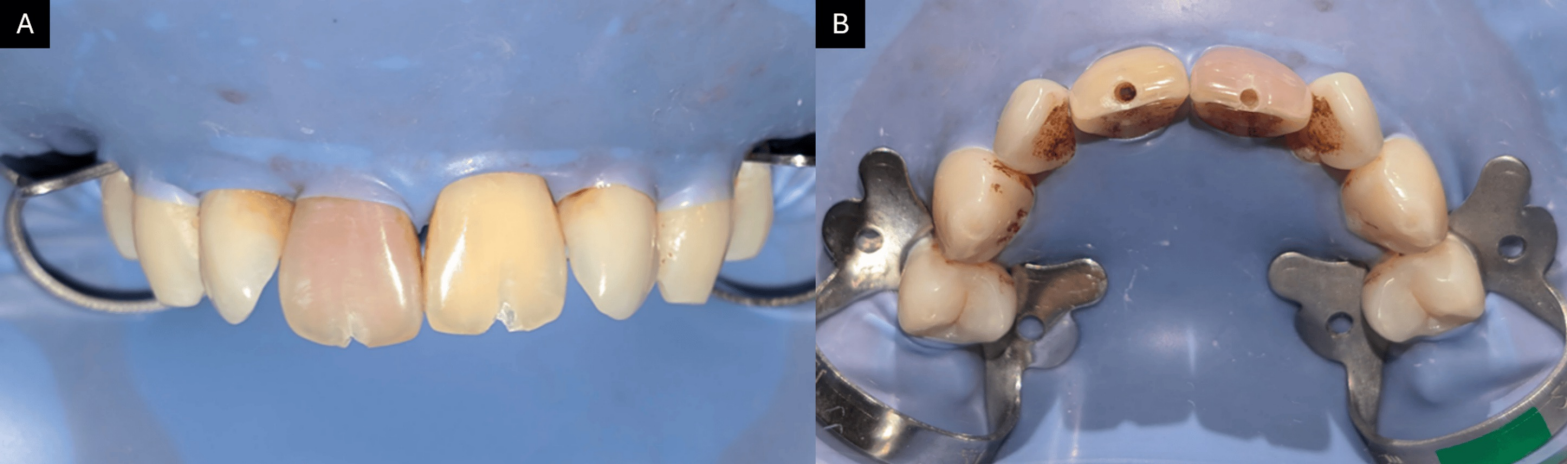
(A) Access opening facial view and (B) access opening incisal view

With the setup complete, local anesthesia was administered to numb teeth 11 and 21, and the teeth were isolated with a rubber dam. The planned data was transferred to the AReneto® system. The augmented reality (AR) glasses were worn to visualize the preoperative access cavity guide plan (Figure 4), and access opening was performed on both teeth (Figure 5).

**Figure 6:**
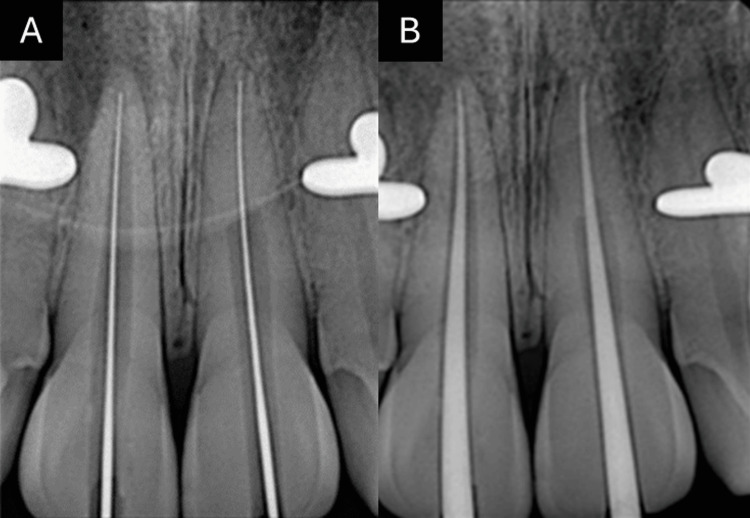
(A) Working length radiograph and (B) master cone radiograph

The working length was determined using an electronic apex locator and confirmed with radiographs (Figure 6, Panel A).

Rotary files (HyFlex EDM, Coltene, 25 mm, Altstätten, Switzerland) were used for the biomechanical preparation of the canals. Irrigation was performed using 5.25% sodium hypochlorite and saline, enhanced with passive ultrasonic activation. After irrigation, the canals were dried. Calcium hydroxide was placed as an intracanal medicament, and the teeth were temporarily sealed. The patient was scheduled for a second appointment in two weeks.

**Figure 7:**
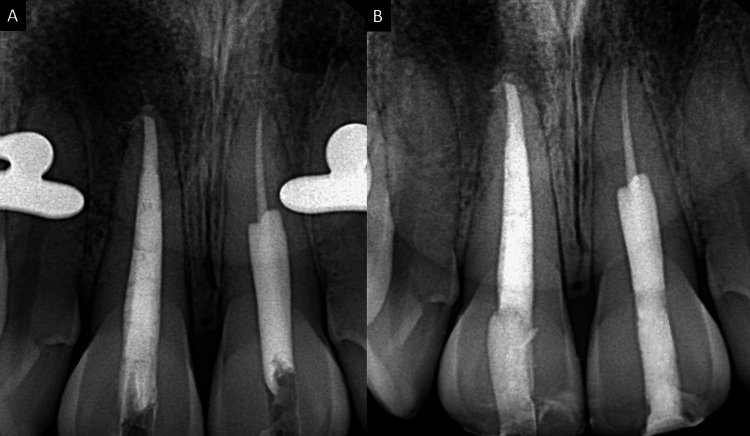
(A) Thermo-plasticized obturation and (B) post-endodontic composite restoration

At the second appointment, irrigation was performed, the canals were dried, the master cone fit was verified (Figure 6, Panel B), and the canals of teeth 11 and 21 were obturated using the thermo-plasticized obturation technique (Figure 7, Panel A).

**Figure 8:**
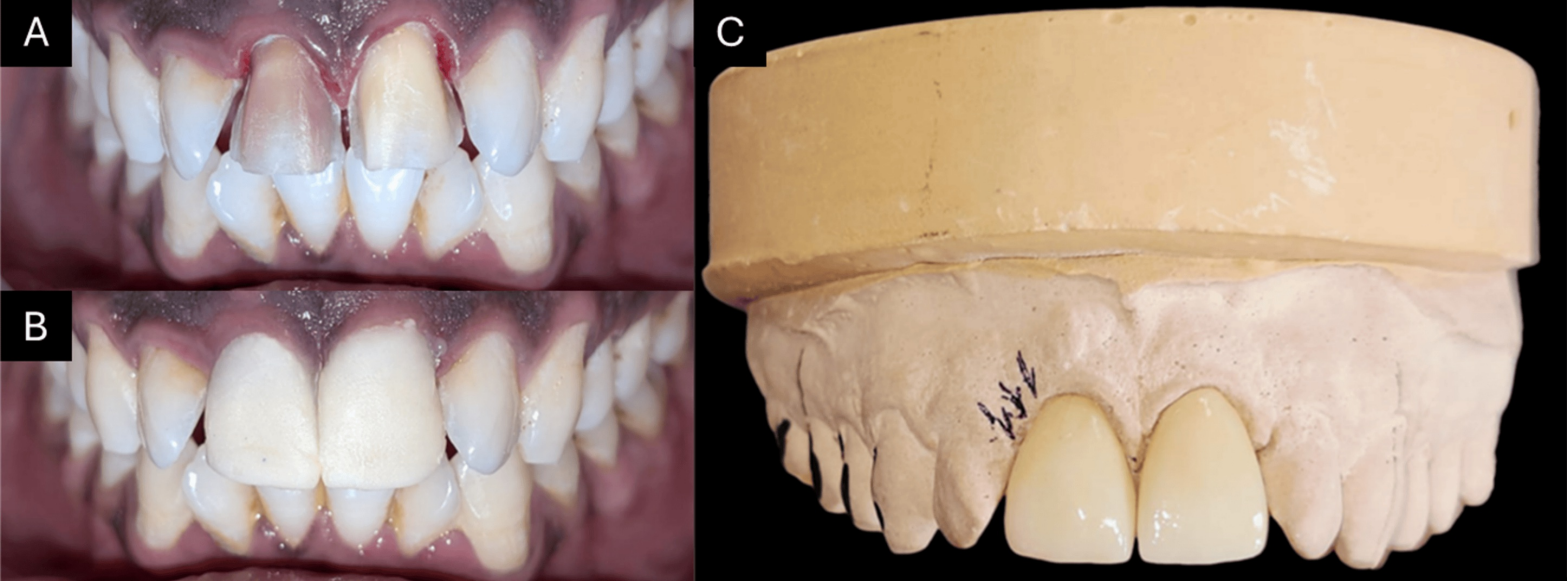
(A) Veneer preparation, (B) temporization, and (C) veneers on cast

One week after obturation, the access openings were sealed with composite resin (Figure 7, Panel B). To enhance aesthetics, veneer tooth preparation was performed (Figure 8, Panel A), and an impression was recorded using additional silicone impression material (Zhermack Elite HD+, Italy, Putty and Light Body) using the single-step putty wash technique. The shades of the teeth were selected and then temporized till the final prosthesis was delivered (Figure 8, Panel B).

**Figure 9:**
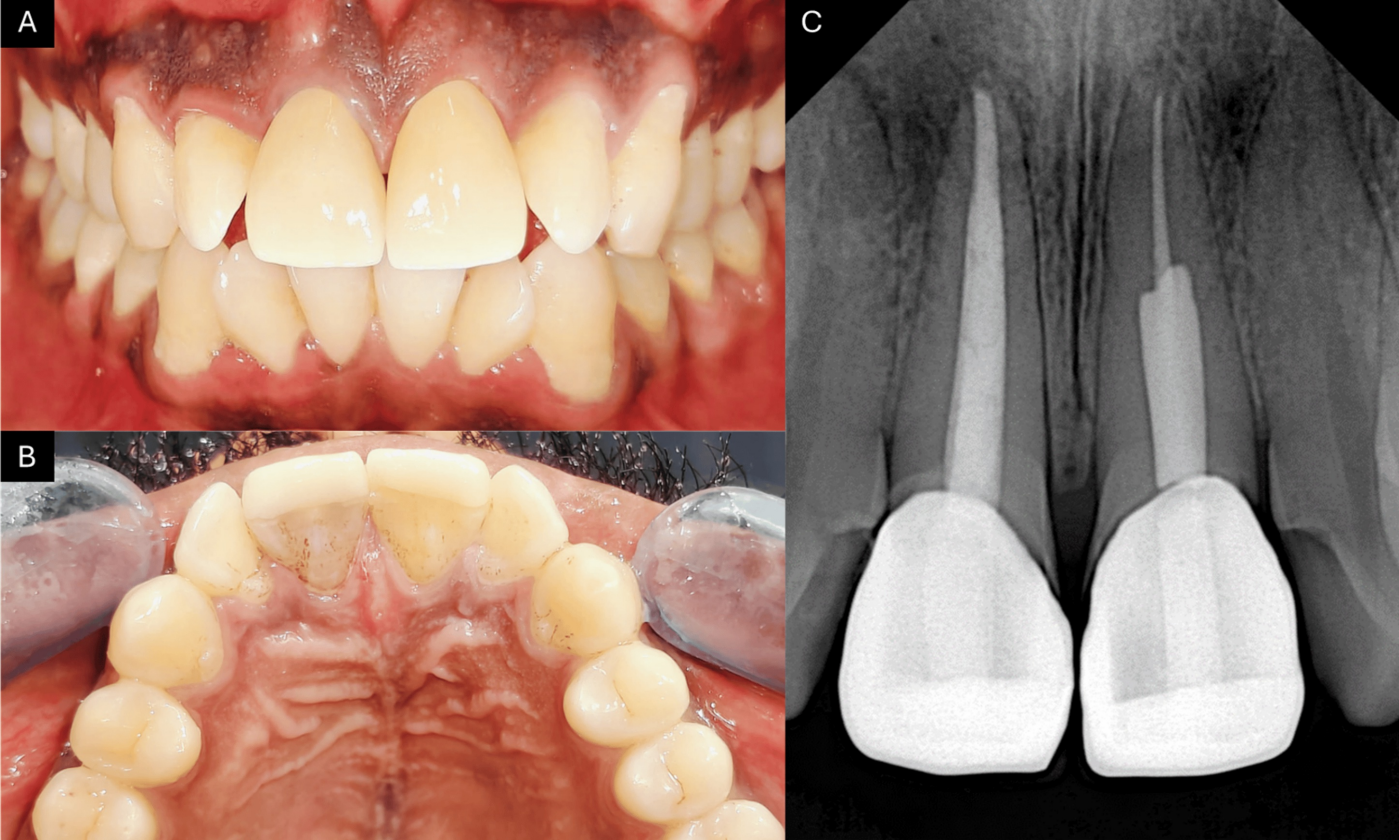
(A) Postoperative facial view, (B) postoperative incisal view, and (C) six-month follow-up radiograph

A bisque trial was done to confirm the fit and shade of the prosthesis. Upon verification, the final veneers (Figure 8, Panel C) were cemented using self-adhesive resin cement (3M ESPE RelyX U200) after application of etchant (Ivoclar N Etchant Gel) and bonding agent (Ivoclar Tetric N-Bond) to the teeth (Figure 9, Panels A and B). The patient attended regular follow-ups at one, three, and six months, and remained asymptomatic, indicating successful treatment (Figure 9, Panel C).

## Discussion

Trauma to maxillary anterior teeth is a common occurrence that often leads to pulpal necrosis and potential calcification, resulting in tooth discoloration [8]. Accurate diagnosis requires thorough clinical and radiographic evaluation. Thermal pulp testing plays a crucial role in assessing the vitality status of the tooth [9].

CBCT assessment provides a better understanding of tooth morphology and periapical changes, especially in cases of calcified canals and large periapical lesions [10]. This enhanced imaging capability aids in accurate diagnosis and facilitates better treatment planning [10].

In the present case, minimally invasive endodontics was selected since there were no carious lesions, and the teeth were intact. Minimally invasive endodontics preserves more of the tooth's natural structure compared to traditional methods, which often require extensive removal of healthy tissue [11]. This approach reduces the risk of tooth weakening and fracture [11].

To reduce the risk of technical errors and shorten treatment time, a computer-assisted method was developed to accurately locate calcified root canals in a minimally invasive manner, giving rise to the concept of "guided endodontics” [11]. Guided endodontic access preparation can be approached in two main ways: static guidance, which uses a template, and dynamic navigation, which employs markers placed in the patient's mouth along with a camera system [11]. AR is increasingly gaining popularity worldwide, offering the ability to overlay information directly in front of the user's eyes [12].

Merging guided endodontics with AR, Roots to Cusps® Private Limited, based in Bangalore, India, developed the AReneto® system. The AReneto® system projects preoperative virtual planning of the access cavity directly before the clinician's eyes through AR glasses, thereby helping the clinicians focus on the operating area and perform minimally invasive access cavity preparation [7].

HyFlex EDM (Coltene) rotary files were used for biomechanical preparation to ensure effective canal cleaning and shaping. Passive ultrasonic irrigation was employed to activate the irrigants, facilitating comprehensive disinfection and accelerating the healing of periapical infections [13].

Thermo-plasticized obturation was opted for this case due to the wide canal morphology of tooth 11, which was not suitable for single cone obturation, and the slight deviation encountered while negotiating the canal in tooth 21. This technique was chosen to ensure a proper canal seal, prevent voids, and reduce the risk of microleakage and reinfection due to its ease of use compared to the conventional lateral compaction technique [14].

As the patient's primary concern was tooth discoloration and the teeth of interest showed no carious lesions, with a minimally invasive access cavity prepared, we opted for veneers for aesthetic rehabilitation. To preserve the healthy tooth structure, full coverage crowns were deemed unsuitable, as they would require extensive removal of sound tooth material, compromising the tooth's strength, fracture resistance, and structural integrity [15].

Impressions were taken to fabricate the veneers using additional silicone material using the single-step putty wash technique. A bisque trial was conducted to verify the fit and shade of the veneers against the natural teeth. Once confirmed, the final veneers were cemented using self-adhesive cement (3M RelyX U200).

The AReneto® system helps the clinician to perform minimally invasive access openings, potentially improving the long-term prognosis of endodontic treatments. However, its effectiveness largely depends on the clinician's expertise. This system reduces chairside time, minimizes operator fatigue, and provides a more comfortable patient experience [7]. Success in endodontics is achieved once canal patency is obtained. In cases of financial constraints or time limitations, the AReneto® system can serve as an efficient alternative to static guides or dynamic navigation systems. The AReneto® system is cost-effective, requiring only glasses and a computer rather than bulky equipment, and avoids the need for STL files and multiple preoperative impressions, which can be uncomfortable for patients [7].

## Conclusions

In conclusion, the AReneto® system demonstrates significant potential in aiding clinicians to perform minimally invasive access cavities by projecting preoperative guide plans directly onto AR glasses. This case showcases the effective use of the AReneto® system in managing a challenging case involving a severely calcified canal, allowing for precise and minimally invasive access. While the system promises to enhance the long-term prognosis of endodontic treatments, it is currently in its developmental stage and functions as a passive assistant. The success of such procedures continues to depend heavily on the clinician's expertise and experience. As the technology evolves, further advancements will be crucial to fully integrate these tools into everyday dental practice, bridging the gap between emerging technology and clinical application.
